# Rapidly Progressing Refractory Hodgkin Lymphoma: A Case Report and a Possible Explanation

**DOI:** 10.1155/2016/7698624

**Published:** 2016-06-26

**Authors:** Ádám Jóna, Gábor Irsai, Sándor Barna, Gábor Méhes, Árpád Illés, László Váróczy

**Affiliations:** ^1^Department of Hematology, Faculty of Medicine, University of Debrecen, Debrecen 4032, Hungary; ^2^Department of Pathology, Faculty of Medicine, University of Debrecen, Debrecen 4032, Hungary

## Abstract

*Introduction*. Hodgkin lymphoma is a highly curable lymphoid malignancy; however treatment of a significant number of patients remains challenging.* Case Report*. The authors present an unusually rapidly progressing case of refractory advanced stage classical nodular sclerosis subtype Hodgkin lymphoma with unfavorable prognosis. A 66-year-old male patient was refractory for first-line doxorubicin, bleomycin, vinblastin, dacarbazine (ABVD) treatment with persistent disease; therefore physicians changed treatment for dexamethasone, cytarabine, and cisplatin (DHAP) and later ifosfamide, gemcitabine, and vinorelbine (IGEV) regimen. Unfortunately the patient developed acute kidney and respiratory failure and died after 6 months of treatment. Current and retrospective histological examination of the patient's lymph node biopsy, skin lesion, and autopsy revealed the same aberrantly expressing CD4 positive nodular sclerosis subtype Hodgkin lymphoma.* Conclusion*. Aberrant expression of T-cell antigens on the Hodgkin and Reed/Sternberg cells could be associated with inferior outcome. T-cell associated antigens should be investigated more often in patients not responding sufficiently to treatment and hence treatment should be intensified or targeted therapy (brentuximab vedotin) should be considered.

## 1. Introduction

Current standard treatment of Hodgkin lymphoma (HL) is expected to cure the majority of patients. Patients with advanced stage disease have 5-year overall survival more than 80% [1]. However, treatment of relapsed and primary refractory patients still remains a challenge. Median survival of patients relapsing after second-line, high-dose therapy and autologous stem cell transplantation is less than 3 years [2]. In this paper the authors present a case of a rapidly progressing refractory HL patient and they try to provide a possible explanation for its biological nature.

## 2. Case Report

In August 2013, a 66-year-old male suffering from high blood pressure and chronic obstructive pulmonary disease presented with symptoms of recurrent fever and generalized lymphadenomegaly. Biopsy from the left axillary lymph node revealed classical nodular sclerosis variant of Hodgkin lymphoma (cNS HL). Stage IV/B disease was confirmed with hepatic, spleen, and extranodal (pulmonary) involvement with an unfavorable prognosis (International Prognostic Score 5). The patient had persistent fever during treatment with several lines of antibiotics (ciprofloxacin, levofloxacin, moxifloxacin, and piperacillin + tazobactam) and ABVD I/1 (doxorubicin, bleomycin, vincristine, and dacarbazine).

**Figure 1:**
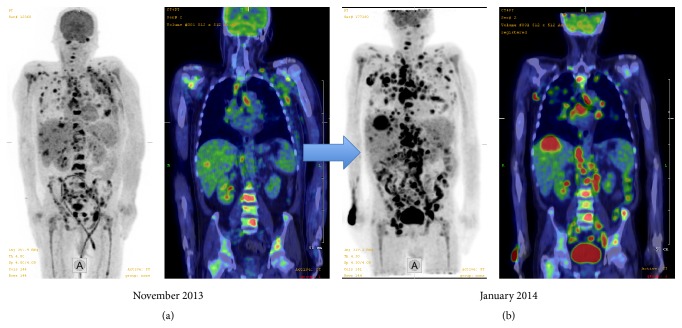
Rapid progression of Hodgkin lymphoma with an extensive disease involving liver, spleen, bone marrow, and lung in a 66-year-old male (a) before DHAP (dexamethasone, cytarabine, and cisplatin) and (b) after 2 cycles of DHAP.

The patient arrived to our center in October 2013 with fever and clinical symptoms of extensive pneumonia. Inflammatory involvement in the left axillary region was also present. As for microbiological specimens,* Candida crusei* was cultured from the bronchial secretion, but hemocultures and skin cultures remained negative. Endocarditis was excluded with transthoracic and transesophageal echocardiography. Cytomegalovirus and cryptococcus serology were both negative. A vertebral magnetic resonance imaging scan was performed due to walking impairment, which revealed tumorous bone involvement, but showed no evidence of infection (either abscess or osteomyelitis). Though the patient received treatment with broad-spectrum antibiotics and antifungal agents (meropenem + moxifloxacin and fluconazole and then amphotericin B), fever persisted as a symptom of progressing HL. Therefore ^18^FDG-PET/CT (^18^fluoro-deoxy-glucose-positron emission tomography combined with computed tomography) was performed in November 2013. The scans revealed an extensive disease with supra- and infradiaphragmatic bone marrow and extranodal involvement (Figure 1(a)). As only half cycle of ABVD was administered, we considered the patient refractory due to his rapidly worsening clinical condition; therefore we decided to change to salvage therapy.

After the first cycle of DHAP chemotherapy (dexamethasone, cytarabine, and cisplatin), a significant response was observed, the patient became afebrile, tendency of inflammatory parameters improved, and improving overall clinical condition was seen. Before the second cycle of DHAP in December 2013, fever reoccurred without any known infectious origin but chemotherapy improved his clinical condition. A subsequent interim ^18^FDG-PET/CT and biopsy of the axillary skin lesion was performed. Unfortunately, ^18^FDG-PET/CT showed rapid progression (Figure 1(b)) in early January 2014.

In the meantime, histopathological analysis of the axillary skin lesion biopsy was performed. The specimen was obtained from the disseminated cutaneous lesion localized on the left side of the thorax. Histologic findings included perivascular infiltration by atypical cells with prominent nuclei, large cytoplasm, altogether resembling blast-like tumorous infiltration. Based on immunohistologic characteristics of strong CD30 positivity, coexpression with CD3/CD4 antigens, light EMA positivity, CD20 and Alk-1 negativity, and 15% Ki-67 proliferation rate with no apparent cells of the Reed-Sternberg morphology, possibility of a peripheral T-cell lymphoma was considered. It was noted, however, that the size and quality of the biopsy were insufficient for proper diagnosis.

Considering these results, the patient was treated with IGEV protocol (ifosfamide, gemcitabine, and vinorelbine). Antibody drug conjugate brentuximab vedotin was considered and was under review for approval by the insurance company, due to CD30 positivity of the tumorous cells in both samples. Unfortunately the patient died in January 2014, during an IGEV chemotherapy cycle from acute kidney failure and respiratory insufficiency.

## 3. Autopsy Results

According to autopsy results, bronchopneumonia was marked as direct cause of death. The autopsy revealed enlarged, firm lymph nodes in the paraaortic, mediastinal, retroperineal, and small pelvic regions. Lungs on both sides were infiltrated by firm, grey nodules with an average diameter of 2.5 cm. Hepatosplenomegaly with bone marrow suggesting hypercellularity was also noted.

Postmortem sampling included skin lesions found on the left thorax, axillary lymph nodes, lungs, spleen, liver, and bone marrow. Routine haematoxylin eosin staining revealed diffuse tumorous cell infiltration in all affected organs. The presence of Reed-Sternberg with lacunar type morphology was obvious, presenting with collagen bands surrounding nodules specifically in the lungs, lobated nuclei, small lobes, less prominent nucleoli, and wide cytoplasm.

Due to the unusually rapid progression of the disease, we decided to perform a wider range of immunostainings. As the direct cause of death was bronchopneumonia, we performed PAS special staining along with immunohistochemistry for CMV antigen presence. CMV pneumonia was excluded due to CMV antigen negativity. Naphthol-AS-D-Chloroacetate Esterase (NASD), CD3, CD4, CD20, CD30, PAX5, LCA, Ki-67, p53, LMP-1, and Bcl-6 stainings were also performed from the bone marrow and lymph nodes as well as the skin lesion.

**Figure 2:**
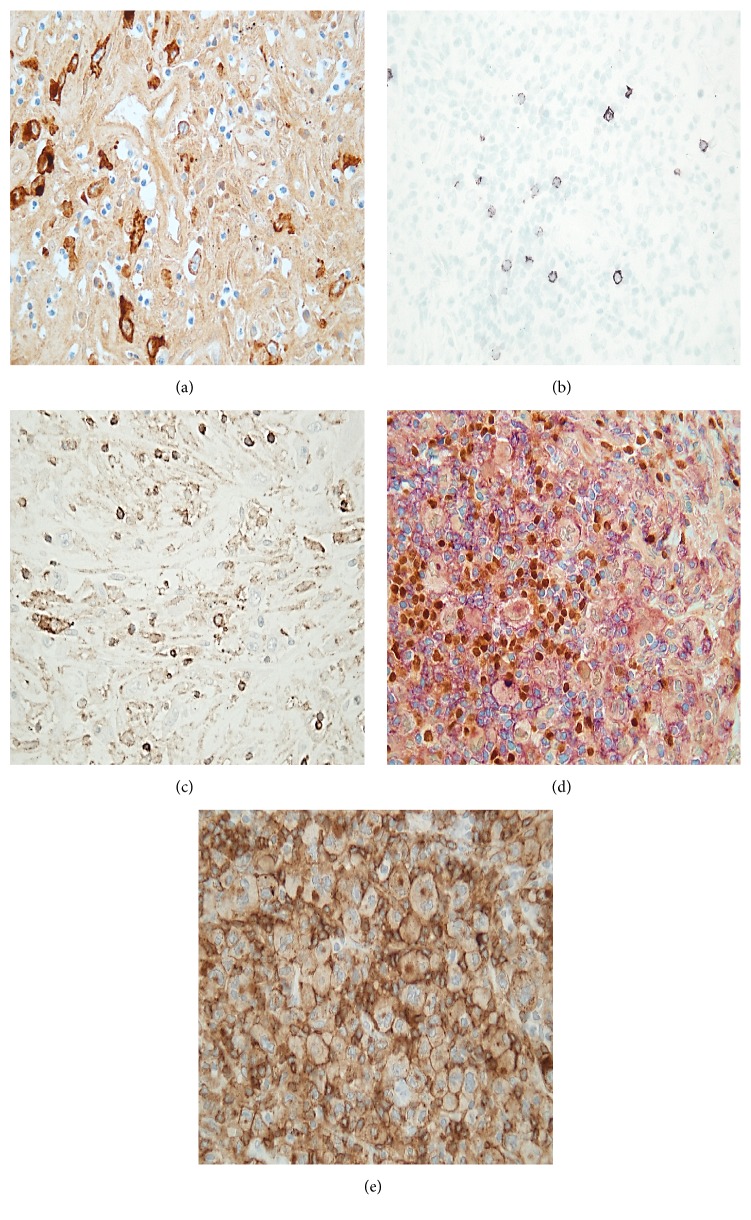
CD30 positivity of the HRS cells in the skin lesion (a), sporadic CD20 positivity in the skin lesion (b), leukocyte common antigen (LCA) negative tumorous cells in the dermis (c), PAX5/CD4 coexpression in the HRS cells from the initial axillary lymph node (d), and unusual CD4 immunoreactivity of the HRS cells from the initial axillary lymph node (e).

Tumorous cells in these regions consequently showed strong CD30 positivity (Figure 2(a)) with CD20 negativity (Figure 2(b)). There was no LCA immunoreactivity amidst the tumorous cells (Figure 2(c)). CD4 immunoreactivity, however, was undeniably positive (Figures 2(d) and 2(e)). A cut-off value of 10% for CD4 positivity was considered acceptable for our immunohistochemical quantification.

Based on our histological findings, we concluded that it was a rare form of CD4 positive Hodgkin lymphoma of the nodular sclerosis variant, with fast progression and resistance to conventional chemotherapy.

## 4. Discussion

Early identification of patients with therapy refractory HL is crucial. Besides the role of interim ^18^FDG-PET/CT, there is increasing evidence for the role of biomarkers. High TARC (thymus and activation-regulated chemokine) level at baseline showed poor prognosis in a multivariate model to response to therapy at baseline evaluation [3]. Soluble CD163 antigen in the serum has also been reported to be consistent with TARC level and response to treatment [4].

In our case, it was not possible to evaluate disease due to its rapidly progressing nature; however, diagnosis of HL was questioned several times. Revision of the original FFPE (formalin fixated, paraffin-embedded) blocks was performed. Retrospectively, both samples revealed the same CD30 positive cNS HL, as did the autopsy; thus involvement of anti-CD30 antibody drug conjugate brentuximab vedotin was established [5]. Unfortunately, State Insurance grants brentuximab vedotin individually, which takes several weeks in Hungary.

There may be other biological markers, which could predict the progression of an otherwise potentially curable disease. Although HL is of B-cell origin, Hodgkin/Reed-Sternberg (HRS) cells infrequently express B-cell markers, although T-cell marker expression is also infrequently reported [6]. When HRS cells aberrantly express T-cell markers, they are very rarely of T-cell origin. When T-cell receptors were first analyzed on a larger cohort of specimens, only 5% of HL cases expressed TCAs (T-cell antigens) on HRS cells. CD2, CD3, CD4, CD5, and CD8 play an important role in T-cell receptor signaling. It is still under debate whether TCA expression might be induced by aberrant microenvironment or by preventing the defective B-cell receptor expression and signaling cascade [7]. In line, by losing B-cell phenotype, silencing of the B-cell program may provide a rationale for HRS cell survival, which may be contributed to TCA expression. Other than CD3 antigen, TCAs are infrequently investigated in routine HL cases. A recent publication has revealed an association of the expressions of these antigens on the surface of HRS cells with worse clinical outcome [8]. Though our case suggests the latter, it is of rapid progression and unfavorable outcome with unusual TCA expression; further characterization of such cases and correlation with their unique antigen presentation has to be cleared.

A physician and a consulting pathologist may decide to further investigate a patient's specimen with aberrant TCAs or cytotoxic markers when the patient is not responding sufficiently to treatment according to interim PET/CT evaluation. When a patients' response is unclear (besides available biological markers such as TARC and sCD163), CD4 positivity may contribute to a therapeutic decision to choose high-dose therapy and autologous stem cell transplantation. Being a relatively cheap technique, aberrantly expressed T-cell antigens, particularly CD4, should be examined at baseline for every nodular sclerosis subtype classical Hodgkin lymphoma patient. Aberrantly expressing CD4 positive nodular sclerosis subtype classical Hodgkin lymphoma patients at baseline may be candidates for frontline brentuximab vedotin administration, if verified in large clinical trials.

## Supplementary Material

Supplemental Figure 1 The cells with HRS morphology showing CD30 expression represented pale nuclear positivity with PAX5 staining compared to the expression in the reactive cells. Pale, reduced PAX5 expression is typical in Hodgkin lymphoma cells. The pale, less intensive PAX5 expression with very intensive CD4 costaining is less clear on our initial and then repeated efforts. Here we provided separate pictures of PAX5 staining to confirm that the cells in questionare of B cell origin. In these cases the stainings were not combined, therefore the reactions are clear to see. We attach the CD4 staining separately with similar dispersion. (a): The HRS cells (←) are negative with CD20 staining, reactive B cells express the staining(<). (b): The HRS cells (←) show pale PAX5 staining, reactive B cells express intensive PAX5 positivity (<).(c):: Reactive cells as well as HRS cells express CD4 staining (←).
