# Supplementary figures and images for: Rapidly Progressing Refractory Hodgkin Lymphoma: A Case Report and a Possible Explanation
Source: Case Rep Med. 2016 Jun 26;2016:7698624. doi: 10.1155/2016/7698624 (PMC4939189; doi:10.1155/2016/7698624)

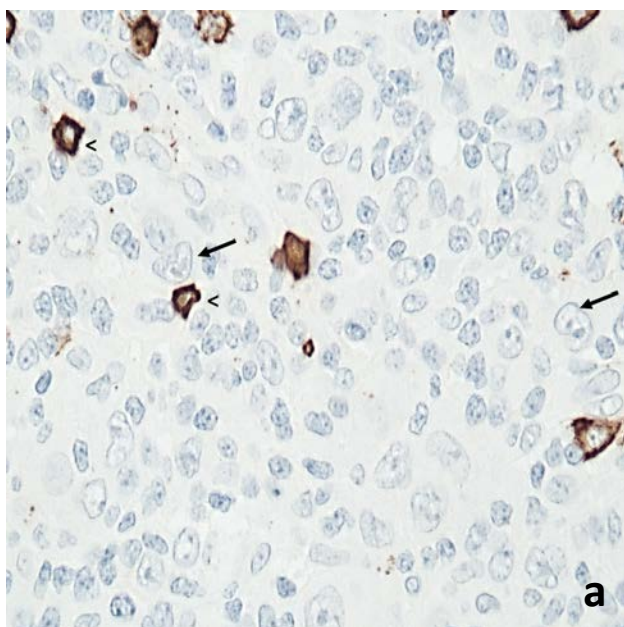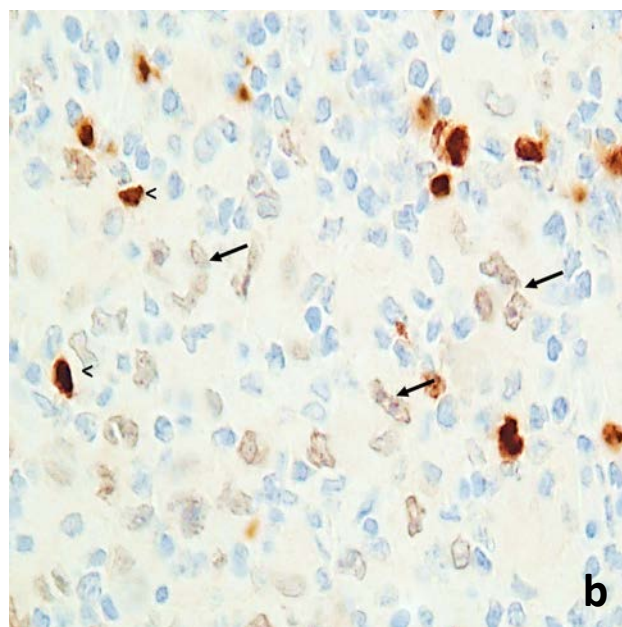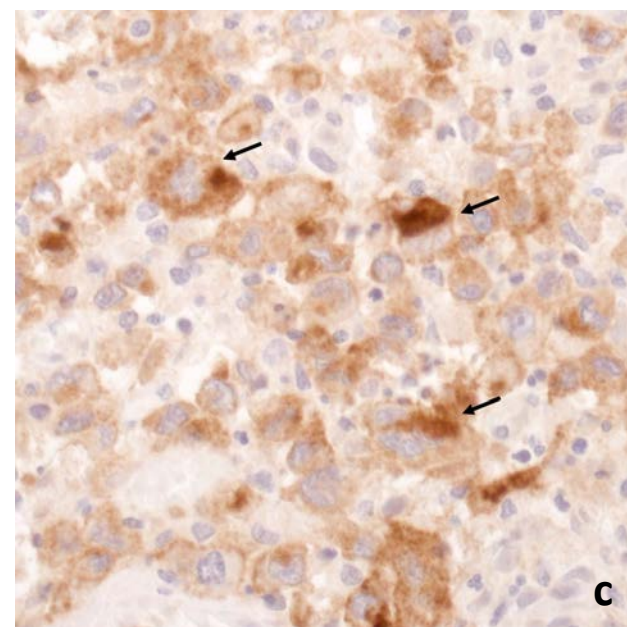

**Supplemental Figure 1**

Supplement: Supplementary file 1 — Supplemental Figure 1 The cells with HRS morphology showing CD30 expression represented pale nuclear positivity with PAX5 staining compared to the expression in the reactive cells. Pale, reduced PAX5 expression is typical in Hodgkin lymphoma cells. The pale, less intensive PAX5 expression with very intensive CD4 costaining is less clear on our initial and then repeated efforts. Here we provided separate pictures of PAX5 staining to confirm that the cells in questionare of B cell origin. In these cases the stainings were not combined, therefore the reactions are clear to see. We attach the CD4 staining separately with similar dispersion. (a): The HRS cells (←) are negative with CD20 staining, reactive B cells express the staining(<). (b): The HRS cells (←) show pale PAX5 staining, reactive B cells express intensive PAX5 positivity (<).(c):: Reactive cells as well as HRS cells express CD4 staining (←). [file 7698624.f1.pdf]
